# The effect of water temperature on routine swimming behaviour of new born guppies (*Poecilia reticulata*)

**DOI:** 10.1242/bio.20149829

**Published:** 2015-03-06

**Authors:** Maud Kent, Alfredo F. Ojanguren

**Affiliations:** Centre for Biological Diversity, Scottish Oceans Institute, University of St Andrews, KY16 8LB, Scotland, UK

**Keywords:** *Poecilia reticulata*, Routine swimming, Temperature, Thermal range

## Abstract

Guppies have successfully established populations in places with thermal regimes very different from the Tropical conditions in their native range. This indicates a remarkable capacity for thermal adaptation. Given their vulnerability to predation as juveniles, acute changes in temperature, which can alter predator-prey relationships, can impact juvenile survival and have amplified consequences at the population level. To understand how temperature may impact juvenile survival and gain insight into their success as an invasive species, we researched the effect of acute temperature changes on the routine swimming behaviour of juvenile guppies. Using a novel 3-dimensional tracking technique, we calculated 4 routine swimming parameters, speed, depth, and variation in speed or depth, at 6 different test temperatures (17, 20, 23, 26, 29, or 32°C). These temperatures cover their natural thermal range and also extended past it in order to include upper and lower thermal limits. Using model selection, we found that body length and temperature had a significant positive relationship with speed. Variation in speed decreased with rising temperatures and fish swam slightly closer to the bottom at higher temperatures. All juveniles increased variation in depth at higher temperatures, though larger individuals maintained slightly more consistent depths. Our results indicate that guppies have a large thermal range and show substantial plasticity in routine swimming behaviours, which may account for their success as an invasive species.

## INTRODUCTION

Temperature can affect every aspect of the physiology and performance of organisms (Johnston and Bennett, 2008; Angilletta, 2009). In particular, the thermal conformity of ectotherms renders them especially susceptible to changes in environmental temperatures (Huey, 1982; Atkinson, 1994). Physiologically, temperature can affect muscle fibre number (Wilkes et al., 2001) and muscle performance (Putnam and Bennett, 1982), endurance (Ojanguren and Brañta, 2000), growth (Angilletta et al., 2004), metabolic rate (Das and Das, 1982), heart rate (Richards, 1963), immune functioning (Maniero and Carey, 1997; Le Morvan et al., 1998) and size and age at maturity (Angilletta and Dunham, 2003). Behaviourally, temperature can elicit thermoregulatory and avoidance behaviours such as altered distribution patterns (Walther et al., 2002), microhabitat use (Taylor, 1988; Adolph, 1990), foraging tactics (Persson, 1986; Fraser et al., 1993; Ayers and Shine, 1997), and courtship behaviours (Hilder and Pankhurst, 2003; Denoël et al., 2005).

The relationship between body temperature and measures of performance is often modelled using thermal performance curves (TPCs, Huey and Stevenson, 1979; Kingsolver et al., 2001; Angilletta, 2006). For most ectotherms, TPCs take a similar shape with performance gradually increasing from a minimum critical temperature to an optimal temperature. After a peak or plateau at optimal temperatures, performance tends to rapidly decline to a critical thermal maximum where performance is zero (Huey and Kingsolver, 1989). These minimum and maximum critical temperatures represent the range of temperatures over which an organism can perform a certain function. The shape and position of TPCs can be influenced by an organism's environment or thermal experience, although there is competing evidence over whether this is due to acclimation, involving phenotypic plasticity (Leroi et al., 1994; Wilson and Franklin, 2002), or adaptation, involving changes in gene frequencies (Zamudio et al., 1995). For instance, Schaefer and Ryan (Schaefer and Ryan, 2006) found that zebra fish reared in variable thermal environments had larger tolerances than conspecifics reared in more stable thermal environments as the result of developmental plasticity and non-genetic adaptation. Despite this and other evidence that the shape and position of TPCs can be moderated through plasticity during an individual's lifetime (Hamdoun et al., 2003; Kingsolver et al., 2004), research also exists showing inheritance and correlations to ancestral conditions (Morrison and Milkman, 1978; Huey and Kingsolver, 1993; Wiens and Graham, 2005). Overall, TPCs are useful tools for predicting an organism's vulnerability to environmental changes (Huey et al., 2012).

To generate accurate TPCs, research looking into the direct impacts of temperature on behaviour and physiology during different life history stages is vital. Specifically, the impacts of temperature on the early life stages of fish are significant since any changes in survival subsequently affect recruitment and can have amplified consequences at the population level (Houde, 1987). By influencing growth and development, temperature affects vulnerability to predators. For instance, at colder temperatures when growth rates often decline, fish remain within the “window” of vulnerability for longer periods of time (Cowan et al., 1996). Furthermore, since colder temperatures can also reduce escape and cruising speeds (Johnston et al., 2001), fish have a reduced likelihood of surviving predator encounters. Warm temperatures also pose a problem as they often result in faster cruising speeds and therefore increased predator encounter rates (Fuiman and Cowan, 2003). These effects on larval physiology and predator-prey interactions may render temperature a key factor in determining juvenile fitness and survival. Research into the shape and position of TPCs during different life stages can yield valuable insight into this species' capacity for thermal adaptation and survival in locations where they are not endemic.

Guppies (*Poecilia reticulata*) are small freshwater fish native to Trinidad and the North coast of South America although they have successfully established populations in every continent except for Antarctica (Deacon et al., 2011). The thermal regimes experienced by many of these invasive populations differ greatly from the tropical conditions they experience in their native range (Deacon et al., 2011). This, in addition to the fact that temperatures in Trinidad can fluctuate up to 7°C in a 24-h period (Reeve et al., 2014), point at a remarkable capacity for both acute thermal adaptation and long-term thermal adaptation. Looking into the impacts of acute temperature changes on routine behaviour can yield valuable insight into the potentially large impact of temperature on survival and provide a better understanding of the mechanisms enabling their success as an invasive species.

Here, we look at the effect of acute acclimation on routine swimming in juvenile guppies. Using a novel 3D tracking technique, the average depth, speed, and variation in speed and depth were calculated for each individual. Speed refers to distance travelled over time and depth was calculated as distance of the fish from the bottom of the tank. Variation in speed or depth refers to how consistently fish maintained a certain measure of performance over the observation period. These four parameters were used to characterize routine swimming and it was expected that changes in temperature would alter these routine swimming behaviours. By focusing on acute temperature acclimation, this experiment demonstrates how even short-term changes or fluctuations in environmental temperatures might impact juvenile survival.

## Materials and Methods

### Fish care and protocol

The guppies used in this experiment were descendants of fish taken from two different Trinidadian streams: Tacarigua and Tunapuna. Habitat differences between the upstream population of Tunapuna and the downstream population of Lower Tacarigua have resulted in different life history traits, such as size and number of offspring produced, which may account for the population differences in juvenile guppy size used in this experiment (Magurran, 2005).

In both streams, guppies naturally experience temperature fluctuations of over 7°C in a 24-h period (Reeve et al., 2014). Generally, upstream populations such as Tunapuna experience lower maximum temperatures than downstream populations due to increased canopy coverage. The experimental guppies were kept under controlled environmental conditions at relatively constant temperatures ranging from 20°C to 26°C.

For this experiment, juvenile fish were collected from stock tanks containing both males and females as well as from maternity tanks containing single pregnant females. In order to standardize age at testing, juveniles were taken from isolated pregnant female tanks as often as possible, which were inspected on a daily basis. Each day, three juveniles from each population were placed in floating tanks within temperature controlled water baths. Three juveniles from each population were tested at each test temperature for each of the three replicates. Juveniles ranged from one day old to three weeks old and were between 6 mm and 14 mm standard length (hereafter SL, SL±s.d., Tunapuna: 8.0±1.7 mm, Lower Tacarigua: 7.5±1.6 mm). To measure the fish after the swimming trials, we placed each juvenile into a petri dish with a small amount of water and took a vertical picture. The pictures were measured using image analysis software (Image J, National Institutes of Health, USA). Fish were introduced into the water bath when the temperature had reached typical temperatures experienced within the stock tanks or maternity tanks (e.g. between 23°C and 24°C). Over a period of 5–7 h, temperatures were gradually changed to the target test temperatures, which were either 17, 20, 23, 26, 29, or 32°C. Juveniles were given at least 18 h to acclimate to test temperatures before experiments began the next day. Given research showing that guppies can experience daily fluctuations up to 10°C in their native habitats (Reeve et al., 2014), 18 h was deemed to be an appropriate time scale upon which to study the affects of acute daily fluctuations considering that the maximum temperature change in this experiment was 9°C. We had no mortality as a result of temperature manipulations and fish seemed comfortably acclimated to all test temperatures, as demonstrated by normal swimming and feeding behaviours the morning of the experiment. The average±s.d. (range) temperatures maintained during the 18-h acclimatization period for each target temperature were 17.1±0.2°C (17.0–17.4), 19.7±0.3°C (19.4–19.9), 23.0±0.1°C (23.3–23.4), 25.3±0.3°C (25.0–25.6), 29.4±0.3°C (29.2–29.7), and 32.2±0.2°C (32.0–32.4).

**Fig. 1.**
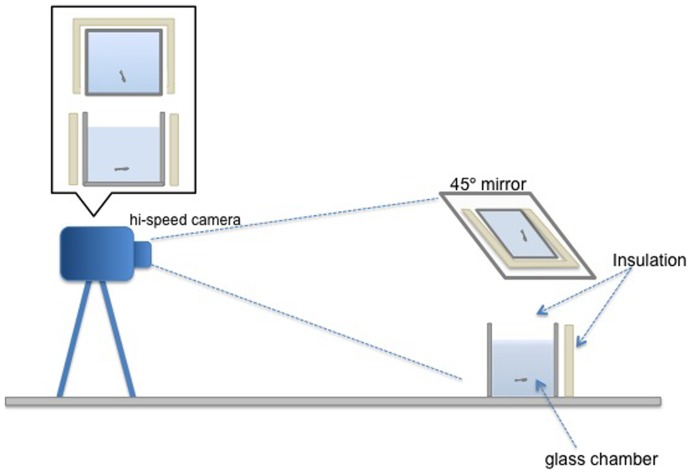
Illustration of experimental setup and apparatus used. A camera was placed 1 m away from a glass tank (10×10×10 cm) placed in a 3-sided Styrofoam insulation chamber with a mirror at 45° overhead. The graph shows 2^nd^ order polynomial trendlines fitted to mean speeds per temperature.

Every day at least an hour before testing began, the fish were fed flake food *ad libitum* to avoid differences in satiation rate that could affect swimming behaviour during video recording. Using water from the water bath to ensure that testing occurred at the appropriate test temperature, fish were placed in 10×10×10 cm glass observation tanks filled to a depth of about 9 mm, with a mirror positioned at 45° overhead. To prevent drastic temperature changes during the filming period, four sides of the glass observation tanks were insulated with polystyrene (Fig. 1). After allowing the fish at least 3 min to get used to the conditions inside the chamber, they were filmed for 10 min with a video camera located approximately 1 m from the observation tank at 30 frames per second. After each trial, fish were photographed and measured for standard length (mm), then placed in stock juvenile tanks.

To analyse routine swimming behaviour, we subsampled each video by taking 1-minute segments from the beginning, middle and end (0–1 min, 5–6 min, and 9–10 min). Each subsampled video was converted into a stack of 60 images (1 frame per second) and imported into ImageJ. Calibrations were then determined for each image stack by dividing a known distance, such as the side of the tank (10 cm), by the number of pixels. The Manual Tracking plugin in ImageJ was then used to obtain X and Y coordinates of fish movement between frames from both the head-on and the overhead views. These coordinates were combined to generate 3D (X, Y and Z) coordinates, which allowed us to calculate average speed (i.e. distance over time) (mm s^−1^), variation in speed, average depth (Z coordinate), and variation in depth.

Fish care and handling complied with institutional and national animal welfare laws, guidelines and policies.

### Statistical analysis

Multiple linear regression models were used to describe the variability of routine swimming speed, depth, and variation in depth and speed based on the potential effects of temperature (T), standard length, Temperature squared (T^2^), and the interaction between T and Length. T^2^ was included in the models to account for a non-linear relationship (SAS Institute Inc., 1999; Montgomery et al., 2012), such as a bell-shaped thermal performance curve (Huey and Stevenson, 1979). Population and replicate, originally included as random effects in this analysis, were removed since there was very minimal variance between groups (replicate variance = 1.7×10^−14^, population variance = 1.7×10^−2^) (Starkweather, 2010). To correct for departures from normality, velocity and variation in velocity were square root transformed and depth and variation in depth were log transformed.

Models were then compared using an information theoretic approach: Akaike's information criterion for finite samples (hereafter AICc). Both delta AIC (Δ_i_) values, a measure of each model relative to the best model, and model weights (w_i_), a measure of the evidence supporting a specific model, were used for model comparison and selection. When there were multiple models with Δ_i_<2, selection was based on the difference in parameters present in each model, reduction in deviance and log-likelihood values (Burnham and Anderson, 2002).

## RESULTS

**Fig. 2.**
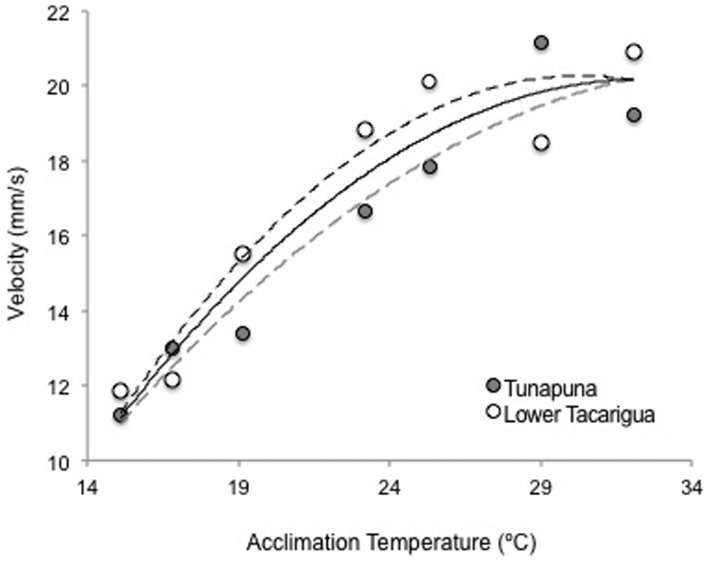
Graph of average swimming speed (mm s^−1^) across all replicates against acclimation temperature by population. The graph shows 2^nd^ order polynomial trendlines fitted to mean speeds per temperature.

A series of six models were tested for their effects on speed, variation in speed, depth, and variation in depth. The variables tested within the set of models included temperature, temperature squared (T^2^), standard length, and an interaction between temperature and length. When testing these models against speed (Table 1), model 2 had the lowest Akaike's information criterion for finite samples (AICc) and received a weight of 0.49. The other top model, model 1, had a Δ_i_ value of 1.39 and weight of 0.25. Both model 1 and 2 include length and temperature, though model 1 also allows for a non-linear relationship through the inclusion of T^2^. Given that both models have essentially the same log-likelihood value (−116.9 vs. −116.5), the additional variable, T^2^, adds little to the top model and can be considered an uninformative parameter (Burnham and Anderson, 2008; Arnold, 2010). Within the top model, both length and temperature are significant (F_(2,110)_ = 21.04, p-value<0.001). Increases in both these variables resulted in corresponding increases in swimming speed (Fig. 2). Fig. 2 shows average speeds from each population across both replicates against acclimation temperature. Although T^2^ did not add to the best model, the polynomial trendlines on this graph suggest that had higher temperatures been tested, there may have been a drop in performance and more conformity to a typical TPC.

**Table 1.**
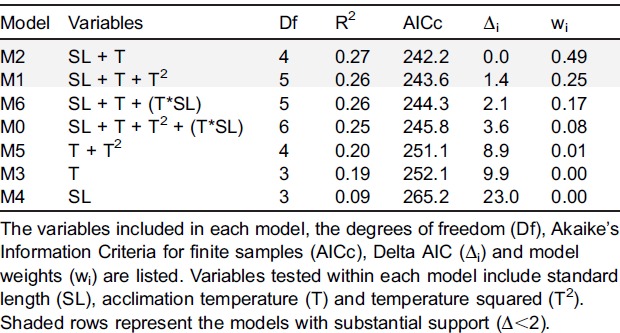
Each model as tested against velocity

For variation in speed, there was substantial support (Δ_i_<2) for models including temperature. Model 5 received the lowest AICc of 477 and a model weight of 0.33. Models 3, 2 and 1 were all within 2 Δ_i_ units of model 5 with AICc scores and model weights of 478 and 0.21 for model 3, 479 and 0.16 for model 2 and 479 and 0.15 for model 1. The two top models, model 5 and 3, included temperature as a predictor of variation in speed. Model 5 only differed through the inclusion of T^2^. Within model 5, however, T^2^ was insignificant indicating that the relationship between variation in speed and temperature is still linear (T^2^: F_(2,110)_ = 5.48, p = 0.09; Temperature: F_(2,110)_ = 5.48, p = 0.05). The 3^rd^ and 4^th^ best models, models 2 and 1, both included temperature and length. The model including only the affect of length (model 4), however, received the highest AICc score and had the least support. No models containing the interaction between temperature and length ranked among the top models. Overall, temperature had a consistent impact on variation in speed throughout all the top models. As temperatures increased, variation in speed decreased. The amount of variance explained by the top models, however, was generally low (R^2^ = 0.08).

For depth, 3 models received Δ_i_ values under 2. The two top models, model 0 and model 6, both included temperature, length, and the interaction between temperature and length. Model 0 only differed through the inclusion of T^2^. Given that T^2^ is not significant in model 0, however, there is reason to select the more parsimonious linear model 6. Furthermore, both these top models had similar weights (0.33 vs. 0.31) and Δ_i_ values (0 vs. 0.2). The third best model, model 2, had much less support with a weight of 0.15 and a Δ_i_ value of 1.6. Temperature had a significant positive correlation to depth with fish swimming slightly deeper as temperatures increased, although there was a large amount of variation in the data (R^2^ = 0.10) (F_(3,109)_ = 4.6, p = 0.04). Length was insignificant (F_(3,109)_ = 4.6, p = 0.11), while interaction between length and temperature was slightly insignificant (F_(3,109)_ = 4.6, p = 0.06).

As with Depth, the top models fitted against variation in depth included the interaction between temperature and length. Model 6 ranked highest with an AICc score of −10.65, a weight of 0.48 and R^2^ = 0.10. Model 0 only just ranked within 2 Δ_i_ units of model 6 (1.6), did not improve upon the R^2^ value (0.09) or the fit of the model (log-likelihood 0.32 lower than top model) and had an AICc score of −9.06 and weight of 0.22. Model 6 and model 0 only differ through the inclusion of T^2^, which was insignificant within model 0, meaning there is justification for selecting model 6 as our best model. Both length and temperature significantly affected variation in depth (length: F_(3,109)_ = 5.23, p = 0.0; temperature: F_(3,109)_ = 5.23, p = 0.01). The interaction between length and temperature was also significant (F_(3,109)_ = 5.32, p = 0.02). While larger individuals varied their swimming depth less than smaller individuals, all juveniles increased variation as temperatures increased.

## DISCUSSION

The results of this study indicate that changes in water temperature, even over a short period of time, can affect the routine swimming activity of juvenile guppies. As test temperatures were increased, average swimming speeds and depths increased while variation in speed decreased, meaning that fish swam progressively closer to the bottom at faster, more consistent speeds as acclimation temperatures increased. The effect of temperature on variation in depth was also found to vary with length, where larger individuals swam slightly shallower than smaller individuals and maintained more consistent depths. The overall impact of temperature on routine swimming activity may be due in large part to the physiological impacts of temperature, although behavioural reasons to modify routine swimming also exist.

The linear relationship found in this experiment between temperature and swimming speed does not conform to the thermal performance curves described in other papers (Randall and Brauner, 1991; Ojanguren and Brañta, 2000; Koumoundouros et al., 2002). Counter to what would be expected in typical TPCs, average speeds did not show any decline at the highest temperatures tested. This could, however, be a reflection of the fact that the upper thermal limits of guppies were not included within the range of test temperatures. Furthermore, although T^2^, the parameter included to account for a non-linear relationship, was not included in the top model against speed, the slight plateau at the highest temperatures may indicate that these were part of the optimal thermal range of guppies and performance would have decreased after this point had temperatures come closer to their upper thermal limits (Fig. 2). Ultimately, the fact that the large range of temperatures tested did not contain the full thermal range of juvenile guppies is indicative of the fact that this species has adapted a wide thermal range that enables them to be effective thermal adaptors and may account for their success as an invasive species. This study also found a remarkable behavioural plasticity in average swimming speeds over a wide range of temperatures, which further demonstrates how guppies can survive in habitats characterized by thermal regimes very different from those experienced in the tropics, or indeed those experienced in their maternal tanks.

Thermal conditions have large physiological impacts on fish and often act as a determining factor in swimming speed (Beamish, 1978; O'Steen and Bennett, 2003). At higher temperatures, studies have shown that fish can maintain faster swimming speeds (Dickson et al., 2002). Increases in maximum velocities after acute acclimation have been attributed to increases in active metabolic rates (Claireaux et al., 2006), cardiac output or oxygen consumption (Beamish, 1970; Clark et al., 2008). Conversely, lower temperatures often result in slower swimming speeds since cold muscle cannot generate the same force as warm muscle (Rome et al., 1990; Green and Fisher, 2004). Importantly, Johnston et al. (Johnston et al., 1990) showed that extended exposure to lower temperatures can result in compensatory mechanisms that allow fully acclimatized fish to out-perform acutely exposed fish. While this study looked into the effect of acute temperature changes, future research could investigate the effect of long-term acclimatization on routine swimming speeds.

Ultimately, alterations in swimming speed could function to behaviourally mitigate the impacts of temperature. Increases in swimming speed in warmer waters, although often physiologically induced, can promote behavioural thermoregulation and enable fish to exploit more optimal thermal niches. For instance, lotic ecosystems, such as those that guppies occupy, are thermally heterogeneous environments that vary both vertically and horizontally. Armstrong et al. (Armstrong et al., 2013) found that juvenile salmon sometimes travel from colder water where they forage into warmer waters where metabolic rates accelerated, promoting faster growth and increased survival potential. In a study by O'Steen and Bennett (O'Steen and Bennett, 2003), in which they found that River barbels reduce activity when temperatures dip below preferred levels, they discuss the potential benefits of reducing activities as a way of conserving energy to perform necessary survival behaviours. At lower temperatures, where maximum capacities are reduced, decreasing speed and swimming less constantly could potentially save energy for escape responses. The behavioural plasticity found in this study could therefore serve to increase juvenile survival rates in the wild and provide a potential advantage to guppies as an invasive species that may experience temperatures radically different to those of their native habitat.

In this study, we also found that swimming speed was affected by juvenile length. We found that larger individuals tended to swim faster than smaller individuals. This positive correlation may be due to the larger propulsive systems (Fisher et al., 2000) or increased anaerobic efficiency (Webb et al., 1984).

Temperature also affected variation in speed. At higher temperatures, juvenile guppies swam at more consistent, higher average speeds. This finding could either indicate a switch to burst-and-coast swimming at lower temperatures or extended periods of inactivity and intermittent movement characterized by a wide range of speeds. Smith and Koenst (Smith and Koenst, 1975) found that juvenile walleye subjected to acute reductions in acclimation temperatures showed decreased swimming activity and periods of idleness. Other studies have observed switches to burst-and-coast swimming at lower velocities when reduced power output reduces performance sustainability and renders burst-and-coast swimming more advantageous (Rome et al., 1984; Rome et al., 1990). This finding, however, has been countered by other studies and the energetics of burst-and-coast swimming are still debated (Kramer and McLaughlin, 2001).

Regardless of whether or not our results point to a switch in swimming mode or periods of inactivity, possible advantages to any type of intermittent locomotion exist. For instance, as muscles fatigue, periods of reduced swimming speed and pauses in active swimming may allow for partial recovery (Kramer and McLaughlin, 2001). Additionally, pauses in active swimming could act to enhance sensory awareness by stabilizing the visual field, reducing motion blur, and allowing time for animals to receive and process all relevant stimuli from their environment (Land, 1999). Behaviourally, varying speed may be advantageous if it reduces conspicuousness of juveniles to predators or increases unpredictability (Humphries and Driver, 1970), especially when maximum swimming capabilities are compromised at lower temperatures. While our results showed an increase in variation in speed at lower temperatures, future research could investigate whether or not burst-and-coast swimming is energetically advantageous in juvenile guppies and therefore a viable reason why intermittent locomotion increased at lower temperatures.

The average swimming depths of juvenile guppies were also found to alter slightly with acclimation temperature. As acute acclimation temperatures increased, juvenile guppies swam slightly closer to the bottom of the tank. In their natural environment, swimming deeper in the water column when thermal conditions exceed optimum temperatures could aid in thermoregulation. In fact, there are many studies (McCullough, 1999; Dunham et al., 2003; Buisson et al., 2008) showing that temperature can have a large impact on the local distribution of fish. For instance, a common response to suboptimal thermal conditions is relocation to thermal refuges, such as areas made shady by undercut banks or protruding vegetation, or areas with cooler water such as side-channels, lateral seeps or groundwater seeps (Bell, 2006; Dallas, 2008). Groundwater outflows in particular provide critical microhabitat through provision of alternative flow regimes, thermal regimes, oxygen and nutrient levels as well as water quality (Heggenes et al., 2010). Bunt et al. (Bunt et al., 2013) found that juvenile Black Redhorse exploit groundwater seepages as thermal refuges and improved water quality. Furthermore, the increased variation in depth found at higher temperatures could suggest that juvenile guppies in this experiment were more active about seeking out potential thermal refuges as temperatures increased. This study may indicate thermoregulatory behaviours in juvenile guppies that would confer survival advantages if employed in their natural habitats.

Overall, this study found that temperature impacts the routine swimming behaviours (such as speed, variation in speed, and depth and variation in depth) of juvenile guppies. As test temperatures were acutely increased, fish swam slightly deeper at faster, more consistent speeds. Our test temperatures, however, did not contain the full thermal range of juvenile guppies, which indicates a wide thermal tolerance. This, in addition to their substantial plasticity in routine swimming behaviours, may be a factor in their success as an invasive species. This study, which investigated the impacts of temperature on routine behaviour, could help expand our understanding of the effect of temperature on juvenile survival and future research could investigate the direct survival implications of the altered behaviours found in this study.

### List of abbreviations

TPC: thermal performance curve; SL: standard length; s.d.: standard deviation; AIC: Akaike's information criterion; T^2^: temperature squared; Δ_i_ values: delta values, a measure of each model relative to the best model; w_i_ values: a measure of the evidence supporting a specific model.
